# When mycologists describe new species, not all relevant information is provided (clearly enough)

**DOI:** 10.3897/mycokeys.72.56691

**Published:** 2020-09-10

**Authors:** Louisa Durkin, Tobias Jansson, Marisol Sanchez, Maryia Khomich, Martin Ryberg, Erik Kristiansson, R. Henrik Nilsson

**Affiliations:** 1 Department of Biological and Environmental Sciences, Gothenburg Global Biodiversity Centre, University of Gothenburg, Box 461, 405 30 Göteborg, Sweden University of Gothenburg Gothenburg Sweden; 2 Department of Forest Mycology and Plant Pathology, Uppsala Biocentre, Swedish University of Agricultural Sciences, Uppsala, Sweden wedish University of Agricultural Sciences Uppsala Sweden; 3 Nofima – Norwegian Institute of Food, Fisheries and Aquaculture Research, P.O. Box 210, 1431 Ås, Norway Norwegian Institute of Food, Fisheries and Aquaculture Research Oslo Norway; 4 Department of Organismal Biology, Uppsala University, Uppsala, Sweden Uppsala University Uppsala Sweden; 5 Department of Mathematical Sciences, Chalmers University of Technology and University of Gothenburg, Göteborg, Sweden University of Technology and University of Gothenburg Gothenburg Sweden

**Keywords:** collaboration, gender equality, metadata, reproducibility, species description, taxonomy

## Abstract

Taxonomic mycology struggles with what seems to be a perpetual shortage of resources. Logically, fungal taxonomists should therefore leverage every opportunity to highlight and visualize the importance of taxonomic work, the usefulness of taxonomic data far beyond taxonomy, and the integrative and collaborative nature of modern taxonomy at large. Is mycology really doing that, though? In this study, we went through ten years’ worth (2009–2018) of species descriptions of extant fungal taxa – 1,097 studies describing at most ten new species – in five major mycological journals plus one plant journal. We estimated the frequency at which a range of key words, illustrations, and concepts related to ecology, geography, taxonomy, molecular data, and data availability were provided with the descriptions. We also considered a range of science-demographical aspects such as gender bias and the rejuvenation of taxonomy and taxonomists as well as public availability of the results. Our results show that the target audience of fungal species descriptions appears to be other fungal taxonomists, because many aspects of the new species were presented only implicitly, if at all. Although many of the parameters we estimated show a gradual, and in some cases marked, change for the better over time, they still paint a somewhat bleak picture of mycological taxonomy as a male-dominated field where the wants and needs of an extended target audience are often not understood or even considered. This study hopes to leave a mark on the way fungal species are described by putting the focus on ways in which fungal taxonomy can better anticipate the end users of species descriptions – be they mycologists, other researchers, the public at large, or even algorithms. In the end, fungal taxonomy, too, is likely to benefit from such measures.

## Introduction

Taxonomy is the science that discovers, identifies, classifies, and describes organisms. Like in any scientific field, the knowledge gained in taxonomy has a value in itself, but it also caters to the needs of other research areas. Almost all studies in biology, and many other sciences, are performed on a taxon (often a species), derivatives from samples of a specific taxon (e.g., a protein), or pertain to the diversity of taxa. This view of the fundamental nature of taxonomy is certain to be shared by scientists and decision makers alike, but surprisingly this is not enough to guarantee a steady long-term supply of resources to taxonomy (Drew 2011). In fact, the funding for taxonomy is at a record low. In what has become known as the “taxonomy crisis” and the “taxonomic impediment” (Wheeler 2004; de Carvalho 2007), taxonomists are finding themselves at nearly the same risk of extinction as the very species they are supposed to study. Various mechanisms have been put forward to visualize and highlight the importance of taxonomy and to give credit to taxonomic work, such as citing authors of species names – and the underlying publications – when using those species names in publications (Wägele et al. 2011). However, the extent to which these suggestions seem to have taken effect appears to be limited, as taxonomy remains locked in a state of crisis (Magoga et al. 2016).

Since the “taxonomy crisis” has been acting out gradually during at least the last 20 years, it is reasonable to think that few biologists are unaware of it. Taxonomists, in particular, are certain to be all too familiar with it, often reporting feeling marginalized in comparison to ecological or molecular initiatives in the context of, e.g., grant writing and scientific funding (Giangrande 2003; Coleman 2015). In this context it is important to focus on the values of taxonomy and how they can be communicated to a wide audience. In this way of thinking, every new species description is a potential outlet for information that is useful not only in taxonomy but also in ecology, conservation biology, agriculture, and so on. The potential of each and every such outlet should be maximized in the interest of taxonomy. But are taxonomic papers written accordingly?

Several of the present authors have spent significant time going through published species descriptions for key data on, e.g., taxonomy, ecology, and geography for compilation into community-driven efforts such as UNITE (Nilsson et al. 2019), PlutoF (Abarenkov et al. 2010), FUNGuild (Nguyen et al. 2016), and Wikipedia (https://www.wikipedia.org/). We have found that scrutinizing species descriptions for key information can be surprisingly frustrating and time-consuming, largely owing to the heterogeneous or implicit ways in which information is sometimes provided (or omitted altogether) in species descriptions. Our experience is that straightforward questions such as “What does the new species do for a living?” are often not addressed at all, or are treated only very indirectly by statements such as “on dead branch of *Quercus*” (perhaps implying a saprotrophic ecology). Another highly relevant question – “Where in the fungal tree of life does the new species belong?” – is similarly often hard to make out from the paper, often requiring a genus name query in NCBI Taxonomy (Federhen 2011) or some similar database. Questions on, e.g., the geographical distribution of the new species are likewise often hard to answer. This lack of information is most unfortunate – certainly, species descriptions should be more or less self-sustained, such that they should not expect significant mycological experience or Google searches on the part of the reader. Our initial observations would indicate that species descriptions are written either for narrow intradisciplinary communication or are disconnected from the wants and needs of many readers. This lowers their impact considerably and is hardly in the interest of taxonomy or mycology at large. Similarly, the field of fungal taxonomy would do good to show that it is, in fact, a vibrant, modern, and collaborative discipline – a discipline that cares little for country borders, where both genders take an active part, and where knowledge is readily shared with, and passed on to, aspiring researchers as well as the public at large. But is that really happening?

To assess whether fungal species descriptions are attuned to both the wants and needs of a target audience beyond taxonomists and the sign of the times, we explored 10 years’ worth of fungal species descriptions of extant mycological taxa in five major mycological journals (plus one botany journal for reference) for a range of factors pertaining to inter- and intra-scientific terms and concepts, science-demographical aspects, and illustrations and visualisations (Tables 1, 2; Suppl. materials 1, 2). We processed the underlying PDF files using a text mining approach where specific keywords were used to simulate a non-taxonomic reader. We also scored each paper manually for a number of features deemed relevant to the overall reader experience – such as the presence of a color photo (or illustration) of the organism being described, whether a map or a habitus photo was provided, and whether we could access the paper from a computer not connected to a university network (Table 2). Our results show that there is much that can be improved in taxonomic descriptions to increase their availability, appeal, and usefulness to a wider scientific and public community and thus the impact of the work and of taxonomy itself. Similarly, fungal taxonomists should adapt their output to an increasing number of automated readers, including data aggregators and search engines. Fortunately, many of these improvements can be implemented in manuscripts in a matter of minutes and at zero cost. Other aspects of our results should make mycologists rethink who should be invited to our studies, and how we would like taxonomic expertise to be passed on to younger researchers. The present study seeks to leave a mark on the way fungal species are described, but we also hope to provide food for thought for editors, reviewers, and members of scientific boards.

## Materials and methods

### Assembly of species descriptions

We went through each issue (2009–2018) of five major, well-reputed mycological journals known to publish new species regularly (Table 1). The journals come from three different continents and are known for their high standards, such that the species descriptions we examined are likely to represent international mycology at its finest. For reference we also included a botanical journal, where we included the fungal descriptions in the fungal description corpus and the plant descriptions in the plant description corpus. All articles whose title made it clear that one or more new species were being described were examined more closely. We retained articles describing at most 10 new species. Efforts such as Fungal Planet (e.g., Crous et al. 2018) – where 100+ species are described in a single paper by 50+ co-authors – were deemed to be too heterogeneous to score meaningfully in a semi-automated context, as we were interested in singular research efforts by coherent groups of taxonomy-oriented co-authors. Descriptions of fossil taxa were excluded. We retained all descriptions of non-fungal taxa (e.g., myxomycetes and oomycetes), but studies where existing species were simply recombined into other genera were excluded. All individual papers that met our criteria were downloaded as PDF files (Suppl. material 2).

**Table 1.** The journals targeted for species descriptions 2009–2018.

Journal name	Journal field	Continent
Mycologia	Mycology	North America
Fungal Biology (Mycological Research)	Mycology	Europe
Mycoscience	Mycology	Asia
Mycological Progress	Mycology	Europe
Studies in Mycology	Mycology	Europe
Plant Systematics and Evolution	Botany	Europe

### Automated and manual examination of the PDF files

The resulting 1,097 PDF files were converted to text using pdftotext version 2.1.4 (https://pypi.org/project/pdftotext/). The text files were mined using a Python script (Suppl. material 1) which searched for the presence of key words and terms related to ecology, geography, taxonomy, molecular data, and data availability (Table 2). In this process, the article titles, author names and affiliations, abstracts, acknowledgements, and literature cited were excluded from the search to reduce the risk of false-positive matches. Out of the 1,097 papers that were scored automatically, we went through 10% manually to verify that the automatic parsing produced reliable results. A number of features relevant to the description of species were not amenable to straightforward algorithmic interpretation – such as the presence of a full-color photo or illustration showing the whole organism being described rather than just micro-morphological details – and these aspects were scored manually by going through each PDF file in Adobe Illustrator CC 2017 (www.adobe.com).

**Table 2.** Estimates obtained by the automated and manual parsing of the PDF files, broken down to three individual years (columns 2–4) as well as overall (column 5). Column 6 indicates our interpretation of the mycological repercussions of the trend in the data. Suppl. material 2 breaks down these estimates onto the individual 1,097 mycological papers, and Suppl. material 1 shows the full syntax used to query the individual papers for each parameter group in column 1. The following estimates are given in per cent. Altitude – altitude of sampling. Biodiversity – whether the term “biodiversity” was mentioned. Climate – whether the word “climate” was used. Climate zone – whether reference to climatic zone was used. Collection – whether reference to “herbarium” etc. was used. Distribution – whether reference to geographical distribution was used. Ecological association – whether any ecological association was indicated. Ecological mode – whether the ecological mode was indicated. Ecology, the term – whether variations of the word “ecology” was used. Family (classification) – whether a family name was provided. GIS/GPS – whether GIS/GPS co-ordinates were provided. Index Fungorum – whether this resource was mentioned. Locality, the term – whether variations of “locality” were mentioned. Molecular availability (TreeBase/Dryad) – whether reference to TreeBase or Dryad was made. Molecular search (BLAST) – whether reference to BLAST was made. Molecular database (e.g., GenBank) – whether reference to, e.g., GenBank was made. Molecular data used – whether DNA data was used. Mycobank – whether Mycobank was mentioned. Order (classification) – whether order was provided. Phylum (classification) – whether phylum was provided. Societal implications – whether societal implications were alluded to. Supplementary data – whether supplementary data were bundled with the paper. Threatened (endangered) – whether the taxon was highlighted as threatened or endangered. Color photo/illustration – whether a depiction of more or less the entire fungus was provided, as opposed to only micromorphological details. Determination key provided – whether a determination key was provided. Discussion section present – whether a dedicated Discussion section was provided. Electron microscopy used – whether electron microscopy was used. Fungal culture shown – whether a photo of a fungal culture was shown. Lead author male – whether the lead author was male. Macro-photo indicates size – whether macroscopic images used scale bars/fiducial markers. Manual micromorphology illustration – papers illustrating micromorphological features using manual illustrations. Map used – whether a map was provided. Paper available – whether the paper was found to be openly accessible. Phylogenetic tree shown – whether a phylogenetic tree was provided (molecular or otherwise). Photo showing biological context – whether a photo or illustration indicating the biological context of the species was provided. Photo of micromorphology – whether microscopic details were illustrated by photos. Spore print provided – whether a spore print was provided. The following estimates are provided as averages. Academic age, last author – the academic age of the last author as assessed through Google Scholar profiles. Academic age, lead author – the academic age of the lead author. Co-authors – the number of co-authors. Co-author continents – the number of continents in the list of co-authors. Co-author countries – the number of countries in the list of co-authors. Co-author departments – the number of departments in the list of co-authors. Female co-authors – the number of female co-authors. Pages – the number of pages. Statistical figures – the number of statistical figures/data visualizations.

Parameter group (automated search)	2009	2013	2018	All years	Trend interpretation
Altitude	26.76	13.64	19.30	16.86	Unclear
Biodiversity	12.68	22.73	27.19	23.25	Positive
Climate	7.04	10.91	18.42	13.67	Positive
Climate zone	88.73	83.64	92.98	89.15	Positive
Collection (specimen/culture repository)	76.06	85.45	92.11	84.59	Positive
Distribution (geography)	74.65	78.18	92.11	82.77	Positive
Ecological association	92.96	75.45	93.86	88.15	Unclear
Ecological mode	77.46	69.09	82.46	75.39	Positive
Ecology, the term	29.58	45.45	56.14	42.02	Positive
Family (classification)	64.79	74.55	85.96	75.39	Positive
GIS/GPS	8.45	2.73	4.39	4.47	Negative
Index Fungorum	4.23	6.36	14.91	11.12	Positive
Locality, the term	29.58	42.73	37.72	35.64	Unclear
Molecular availability (TreeBase/Dryad)	33.80	50.91	69.30	53.78	Positive
Molecular search (BLAST)	9.86	34.55	46.49	35.46	Positive
Molecular database (e.g., GenBank)	57.75	84.55	94.74	85.69	Positive
Molecular data used	71.83	90.91	97.37	90.79	Positive
Mycobank	53.52	94.55	92.11	88.06	Positive
Order (classification)	57.75	64.55	71.93	64.81	Positive
Phylum (classification)	21.13	34.55	50.88	31.91	Positive
Societal implications	50.70	48.18	64.91	53.60	Positive
Supplementary data	5.63	19.09	37.72	24.43	Positive
Threatened (endangered)	0.00	2.73	3.51	2.37	Positive
**Parameter group (manual search)**					
Colour photo/illustration	30.99	70.91	88.60	73.02	Positive
Determination key provided	29.58	27.27	18.42	24.52	Negative
Discussion section present	71.83	75.45	79.82	77.58	Positive
Electron microscopy used	23.94	26.36	22.81	24.89	No change
Fungal culture shown	22.54	21.82	34.21	25.16	Unclear
Lead author male	72.73	69.33	60.26	68.73	Positive
Macro-photo indicates size	60.98	58.02	60.64	63.34	No change
Manual micromorphology illustration	40.85	53.64	35.09	42.66	Unclear
Map used	9.86	10.00	7.02	6.38	Negative
Paper available	71.83	74.55	72.81	77.85	No change
Phylogenetic tree shown	61.97	84.55	93.86	84.59	Positive
Photo showing biological context	52.11	59.09	71.93	62.26	Positive
Photo of micromorphology	81.69	68.18	72.81	74.20	Unclear
Spore print provided	0.00	0.00	0.00	0.09	No change
**Averages (manual search)**					
Academic age, last author	29.47	30.66	28.00	27.99	Unclear
Academic age, lead author	23.11	20.65	12.30	18.11	Positive
Co-authors	3.66	4.18	4.97	4.40	Positive
Co-author continents	1.38	1.41	1.55	1.45	Positive
Co-author countries	1.52	1.77	2.06	1.85	Positive
Co-author departments	2.38	2.95	3.33	2.98	Positive
Female co-authors	0.89	1.06	1.71	1.21	Positive
Pages	9.00	9.75	12.87	10.88	Positive
Data visualizations	0.17	0.13	0.09	0.17	Negative

### Assessment of demographic parameters

The number of co-authors, distinct co-author departments, countries of origin and continents of origin (using the seven-continent system) of the co-authors were counted manually to quantify the extent to which taxonomy is practised as a collaborative pursuit. We sought to establish the gender of all co-authors by brief queries in Google, Google Scholar, and ORCID (https://orcid.org/). Only articles where we could determine the gender of all co-authors were used to infer the proportion of female co-authors and lead male authors. In an attempt at quantifying recruitment of aspiring researchers into taxonomy, we made the admittedly coarse assumptions that the last author was the supervisor, mentor, or taxonomic expert, and that the first author was a student or a nascent taxonomist. Google Scholar was used to determine the academic age of an author: year-of-the-oldest-publication minus year-of-the-most-recent-publication, in a way that dismissed obvious homonyms and ambiguous entries. Unresolved cases were left out from the comparison.

## Results and discussion

For convenience we group our results and discussion under the headings Ecology and geography, Systematics and taxonomy, Metadata and data availability, Visualisation, and Demographical aspects. The overall automated and manual estimates are found in Table 2, whereas the full set of results broken down to each individual paper is found in Suppl. material 2.

### Ecology and geography

Most biologists would probably agree that taxonomy should be pursued in light of as many data sources as possible, including molecular, morphological, and ecological information. The output of taxonomic work should similarly be rich and many-faceted. However, the fact that the word “ecology” (and its variations) was used in only 42.0% of the examined studies somehow speaks against this assertion. On a more positive note, explicit reference to host, substrate, habitat, or partner was made in 88.1% of the cases, and a reference to the nutritional mode of the new species was made in 75.4% of the cases. The word “ecology” and any of the 19 other ecology-related keywords (Suppl. material 1) we used were completely absent in 3.2% of the studies (Suppl. material 2), suggesting that only a very small number of species descriptions are nucleated in what seems to be either complete disregard or lack of ecological data, or in total ignorance of the wants and needs of the scientific community.

We acknowledge that when a new species is described, there may be no or limited occurrence data beyond the type locality. Still, variations of the words “distribution” and “geography” were mentioned in a strong 82.8% of the studies. Although explicit reference to variations of “climate” was found in only 13.7% of the studies, a full 89.2% of the studies featured climate-related words such as “temperate” or “tropical”. GIS/GPS co-ordinates were provided in a much more modest 4.5% of the studies, and 6.4% of the studies provided a map. 62 (5.9%) of the studies that did not provide GIS/GPS co-ordinates provided a map instead. A total of 89.9% of the studies provided neither GIS coordinates nor a map, and 64.4% lacked any relevant variation of the word “locality”. This does little to facilitate recollection of the species at the type locality. Altitude/elevation was mentioned explicitly in 16.9% of the studies. It strains credibility that more than 80% of all fungi described during 2009–2018 were collected at sea level, suggesting that the absence of altitude information should not be taken to mean sea level.

62.3% of the studies featured at least one photo or illustration that gave at least some sort of feeling for the biological context of the new species, typically by showing the collection site, the collection spot, or the substrate of collection. We feel that there is room for improvement here, particularly if taxonomy indeed seeks to produce results of relevance and interest that extend beyond the field.

### Systematics and taxonomy

It is surprisingly common to describe a new fungal species without mentioning where in the fungal tree of life it belongs: a phylum-level name was found in 31.9% of the studies; order, 64.8%; and family, 75.4%. The intersection of these estimates was 20.8%. In a few cases, some of this information may be truly unknown for the species being described (e.g., Torres-Cruz et al. 2017), but we argue that phylum, order, and perhaps family assignment is known for the vast majority of fungi being described. By knowing where the new species belongs, but not writing it out explicitly, the underlying authors rely on the reader to fill in the mycological gaps. This strikes us as unfortunate, because non-mycologists as well as automated data extraction tools such as data aggregators and search engine indexation software may lack this expertise. In fact, even many mycologists will probably have to look up where, e.g., the family Sphinctrinaceae belongs. The situation is not alleviated by the fact that Index Fungorum, MycoBank, and NCBI Taxonomy regularly disagree on family- and order-level placement of genera (owing to, e.g., differential updates and taxonomic opinion). Thus, even readers who actively go looking for this information may be left clueless or misinformed. Although the fungal family level is in a state of flux in many parts of the fungal tree of life, we can’t think of any good reason not to mention at least the phylum and order level affiliations of new species. It is thus not surprising that Catalogue of Life (https://www.catalogueoflife.org/) and similar efforts often fall short of providing the precise taxonomic placement of individual species in the fungal tree of life, even when this information was known to the mycologists who described the species in the first place. Clearly, mycology does not stand to benefit from the presence of incomplete taxonomic information in online repositories.

Although taxonomy represents a core aspect of biodiversity, variations of the word “biodiversity” are not commonly used in papers describing new species of fungi – only 23.2% of the studies used it. This comes across as a missed opportunity to place the new species in a richer context – and to have the underlying paper indexed properly in search engines and automated classifiers of scientific papers. Highlighting the importance or relevance of the new species to society (e.g., agriculture, forestry, or biotechnology) – if motivated – would similarly lead to a wider readership and better article indexation. However, a moderate 53.6% of the studies featured such keywords. A much lower number of studies – 2.4% – made a reference to the threatened or endangered nature of the new species or its habitat, although this may be difficult to know at the time of description.

Where is the underlying specimen or culture deposited? We found it quite common (15.4% of the cases) to provide this information in a way that does not employ any variations of the words “herbarium”, “fungarium”, “museum”, or “culture collection” – an example would be “deposited in H”. The reader would then have to know – or find out – that H is a herbarium at the University of Helsinki, Finland. This poses no challenge to a seasoned taxonomist (through recourse to, e.g., Index Herbariorum at http://sweetgum.nybg.org/science/ih/ or GRSciColl at https://www.gbif.org/grscicoll), but we imagine that other readers would struggle with this, as would data mining efforts to extract information from scientific papers. Improving clarity by writing, say, “deposited in herbarium H” – and why not write out the name of the herbarium in full? – should be easy enough. 11.1% of the papers mention “Index Fungorum” (http://www.indexfungorum.org/names/names.asp) and 88.1% “MycoBank” (Robert et al. 2013) explicitly, showing that writing out names in full is common in other contexts.

Identification keys help define what exactly differentiates the new species from others, and 24.5% of the papers we examined featured an identification key. There can be many reasons why a key would be premature or impossible to construct for various fungal taxa, such that whether 24.5% is a comforting estimate or not is hard to say. Determination keys are, however, becoming rarer over time (Table 2).

### Metadata and data availability

**Figure 1.**
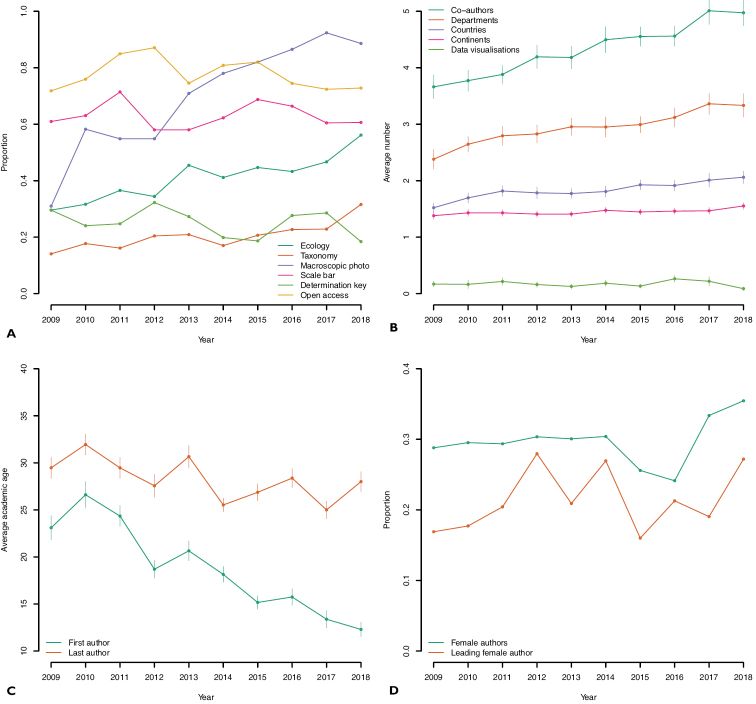
**A** Data and metadata in the description of fungal species 2009–2018. The x axis depicts year and the y axis depicts proportion of studies (from 0 to 1) fulfilling a specific criterion. Dark green – proportion of studies mentioning the word “ecology” or its variations; brown – proportion of studies giving a complete account of the taxonomic affiliation of the new species (family, order, and phylum); purple – proportion of studies with a macroscopic colour photo/illustration of the new species; pink – proportion of studies with macroscopic photos, that also indicate the size of the depicted object through a scale bar or a fiducial marker; light green – proportion of studies with an identification key; yellow – proportion of openly available papers for each year as assessed in 2020 **B** demographical and publication trends showing the average number of co-authors (dark green), departments (brown), countries (purple), continents (pink), and number of data visualizations (light green) over time. The bars indicate the yearly standard error **C** the average academic age of the first (green) and last (brown) co-author over time. The bars indicate the yearly standard error **D** the proportion of female co-authors (green) over time plus the proportion of female lead authors (brown).

The proportion of species descriptions making use of DNA sequence data – as deduced from the use of variations of keywords such as “PCR”, “DNA”, and “sequencing” – is on the rise, from 71.8% in 2009 to 97.4% in 2018 (Fig. 1). Across all years, at least one DNA sequence-related keyword was found in 90.8% of the studies, an estimate that blends well with the 94% reported for fungi by Miralles et al. (2020), who examined papers from 2002, 2010, and 2018. The proportion of DNA-based studies depositing their multiple sequence alignments or phylogenetic trees in TreeBase (Vos et al. 2012) or Dryad (https://datadryad.org/) is similarly on the rise: overall, 53.8% of the studies mention TreeBase/Dryad, up from 33.8% in 2009 but down from the 69.3% of 2018. The problem of poor data availability in systematic mycology has been made apparent before (Nilsson et al. 2011; Drew et al. 2013), so we were happy to note a gradual – albeit slow – improvement over time. If taxonomy is to be a modern, reproducible, integrative, and standards-compliant field (see, e.g., Dimitrova et al. 2019 and the MIAPA standard, Leebens-Mack et al. 2006), then there is simply no excuse for not sharing the underlying phylogenetic – and other – data in standardized ways (Grandcolas 2019; Miralles et al. 2020). Increased data deposition is furthermore likely to improve the visibility of the underlying article and thus taxonomy at large.

To simulate whether the general reader could access the underlying PDF publications by Google searches, we queried Google by pasting the name of the paper in quotation marks and then scrutinizing the first two pages of hits manually (February 2020). We did this from computers not connected to any university network. We accepted hits to PDF files and full-text papers in the HTML format of both the final, published paper and to any preprints in, e.g, bioRxiv (https://www.biorxiv.org/), and we accepted both legal as well as juridically more dubious sources of PDF files. If any sort of registration was needed to access the PDF file, we scored it as “not available”. We found that 77.8% of the studies could be accessed from outside university networks. The observation that more than 20% of the taxonomic output of the mycological community cannot be readily accessed by the general public comes across as unfortunate. However, all of the journals we targeted allow the submission of preprints to online repositories. Thus, submitting a vetted preprint at least post-publication (in order not to confuse effective publication dates of names) is a way around this inaccessibility (cf. Kwon 2020). It is, evidently, a solution that is not being explored to the extent it could have been by the mycological community.

Many cases of taxonomic mistakes, redundant species descriptions, and laboratory contaminations would have been avoided if the authors had subjected the newly generated DNA sequences to a simple BLAST search in, e.g., GenBank (Nilsson et al. 2012). Sadly, only 35.5% of the studies mention BLAST, although the trend is positive (Table 2). A clear majority (85.7%) of the examined studies make explicit reference to one of the INSDC repositories (GenBank, EMBL, and DDBJ), again in a positive trend. Bundling supplementary material with species descriptions is a good way to increase reproducibility and provide additional, helpful information with respect to the new species without consuming valuable page space in print journals. Here we envisage additional photos or drawings of fungal specimens or the collection site, or perhaps extended maps, field notes, or laboratory details. However, a somewhat disappointing 24.4% of the studies saw fit to include at least one supplementary item.

### Visualization

The average study was 10.9 pages long, although we did not correct for the number of described species in each paper. The studies grew more voluminous over time (Table 2), possibly as a consequence of the inclusion of more analyses based on molecular data. A high 73.0% featured at least one color photo or illustration showing more or less the whole fungus being described (in the sense of a full fruiting body rather than only microscopic details or a spore print). Somewhat disappointingly, only 63.3% of the macroscopic photos/illustrations featured a scale bar or a fiducial marker, leaving assessment of size problematical for more than a third. 25.2% of all studies contained at least one photo of a fungal culture, and a spore print was presented in 0.1% of the studies. 98.5% of the studies featured at least one visualization of a micromorphological detail or structure. In total, micro-morphological details were illustrated by line drawings in 42.7% of the studies; photos (74.2%); and electron microscopy (24.9%). 19.2% of the studies featured both a micromorphological photo and a line drawing; a total of 6.2% of the studies commendably used all three techniques. A phylogenetic tree was displayed in 84.6% of the studies. Since 90.8% of the studies used molecular data, this means that a few studies used molecular data without presenting a phylogenetic tree. A cursory look at a few of these indicated that techniques such as RFLP had been used to generate a fingerprint of the new species. The average study featured 0.17 data visualizations (e.g., a graph or a chart other than a phylogenetic tree).

### Demographical aspects

The average number of co-authors was 4.4, which was higher than we expected given that taxonomy is sometimes touted as a solitary discipline. The average number of departments, countries, and continents were 3.0, 1.9, and 1.5 – again higher than we had expected. Plotting the number of co-authors and countries over time (Fig. 1) suggests that fungal taxonomy is slowly becoming an increasingly collaborative and international discipline. It is, however, a discipline dominated by males: out of the 549 papers for which we were able to establish the gender of all co-authors, 30.4% were male-only papers and 3.6% were female-only papers. These 549 papers comprised a total of 2,224 co-authors, of which 662 (29.8%) were female. Males were lead authors in 68.7% of the 758 papers for which we were able to determine the gender of the lead author. Our gender-related estimates certainly leave room for improvement of inclusivity and career opportunities in mycology (cf. Salerno et al. 2019), although they slowly improve over time (Table 2). Our admittedly crude attempts at quantifying the extent to which recruitment of aspiring researchers and the passing on of knowledge is going on in fungal taxonomy showed that the average academic age of the last author (28.0 years) is higher than that of the first author (18.1 years), perhaps hinting that knowledge does seem to be passed on to younger generations (or at least a younger generation) albeit somewhat slowly. One hundred non-taxonomical mycological papers from the same journals produced similar figures – 27 and 16 years, respectively – hinting that this issue may not be specific to taxonomy. In fact, the high academic age of the authors may be part of explaining the gender bias, as gender equality tends to decrease upwards in the academic hierarchy (Potvin et al. 2018).

### Comparison with the botanical species descriptions

Although the key terms and concepts to look for in a mycological species description will be somewhat different from those of a botanical counterpart, we did find some notable differences between the description of fungi and plants. It should be kept in mind that our botanical reference corpus was limited to a single journal and 40 papers, and the extent to which our results can be extrapolated to botany at large remains unknown. Nevertheless, botany comes out on top of mycology when it comes to specifying the type locality through either a map or GIS/GPS co-ordinates: 65% of the botanical studies did this, as compared to only 10.4% of the mycological. On the other hand, the use of molecular data is more widespread in mycological species descriptions (90.8%) than in botanical (60.0%). 59.2% of the mycological studies that relied on molecular data made these available in TreeBase/Dryad, compared to 8.3% of the corresponding botanical papers. Full-color macro-illustrations of the species being described were more common in mycology (73.0%) than in botany (55.0%). The number of co-authors on botanical species descriptions is lower (3.6) than in mycology (4.4), and so is the average number of female co-authors (0.94 vs. 1.2). Mycology comes across as a somewhat more collaborative discipline in that the average number of co-authors from different departments, countries, and continents are all higher in mycology, but botany struggles somewhat less with recruitment of aspiring taxonomists (Suppl. material 2). These figures should be interpreted with the very limited size of the reference corpus in mind, but they do seem to suggest that botany and mycology can learn from each other when it comes to describing new species in the 21^st^ century. Joint meetings such as the 2009 meeting of the Mycological Society of America, American Bryological and Lichenological Society, American Fern Society, American Society of Plant Taxonomists, and Botanical Society of America (http://2009.botanyconference.org/) are commendable in this respect.

### Biases and shortcomings of our approach

The semi-automated nature of our approach is not without potential shortcomings, and we are likely to have both under- and over-estimated some of our parameter values. As an example of an overestimation, a study could mention “DNA” or perhaps “PCR” without actually making use of sequence data in the description of the new species. This would have led us to the incorrect conclusion that DNA sequence data was used in the description of the species. As an example of an underestimation, a study could conceivably provide information on the ecology or nutritional mode of the new species without using any of the ~20 terms we looked for, leading us to the erroneous conclusion that nothing was said about the ecology of the new species. Since we processed nearly 1,100 mycological papers, such outlier cases will not have contributed much to our estimates. Our manual verification of 10% of the papers did not reveal significant cause for concern with respect to over- or under-estimations.

A potentially larger bias lies in our choice of journals. We purposely selected five major international mycological journals with significant impact factors, stringent editorial and review processes, and very detailed author instructions. The journals are not solely focused on taxonomy but cover a wide spectrum of mycological subdisciplines, and the papers published therein can therefore be expected to be geared towards a more general mycological audience. However, fungal species are described also in other outlets. For instance, there are 29 mycological journals with a formal Web of Science impact factor for 2019. Indeed, Schoch et al. (2012) found that the fungal ribosomal nuclear internal transcribed spacer (ITS) sequences in GenBank stemmed from over 500 different scientific journals. Yet other journals would not even be represented in GenBank because they have yet to publish their first species using sequence data (focusing instead on morphology-based descriptions). We speculate that at least some of these other journals may have less stringent editorial and review processes (in fact, several journals that publish new species of fungi do not use formal peer-review at all). Along the same line, many of these journals are not available digitally, and some are printed in black and white. Thus, our estimates pertain to the state-of-the-art species descriptions of fungi rather than the full spectrum of fungal species descriptions. While not all of our estimates are flattering for fungal taxonomy (e.g., Table 2), it is likely that they still paint an overly optimistic picture of fungal species descriptions at large.

### Take-home messages for mycology

The International Code of Nomenclature for algae, fungi, and plants (Turland 2018) stipulates the minimal requirements for publication of new names (species). Notions of, for example, ecology or geographical distributions, or inclusion of illustrations, are not part of those requirements (Seifert and Rossman 2010; Hawksworth 2011). But rather than asking what the minimal requirements are, mycologists should strive to showcase taxonomy as a vibrant, exciting field where species are described in the richest possible way, where all data and metadata elements are machine readable with persistent identifiers, and where ample auxiliary data are posted to online repositories and community initiatives such as Zenodo (https://zenodo.org/), FigShare (https://figshare.com/), Wikimedia Commons (https://commons.wikimedia.org/), and Open Tree of Life (https://tree.opentreeoflife.org/) in standards-compliant formats and data structures (Mons et al. 2017). To some extent, our results question whether this is how fungal species are described at present. The fact that a full 58.0% of the studies did not mention any variations of the word “ecology”, or that 76.8% of the studies lack the word “biodiversity”, is certainly not encouraging, for instance. We argue that fungal species should be described in the richest possible way; in fact, a moderate 77.6% of the studies featured a formal “Discussion” section, which for the remaining studies seems like missed opportunities to anchor the new species in a richer mycological and biological context. With an average of 4.4 co-authors on the 1,097 papers, there would clearly be room to invite one or more additional co-authors to add, e.g., ecological aspects to the description. Similarly, many opportunities to bundle helpful supplementary data are waiting to be filled (75.6% of the studies did not bundle any supplementary material). We do realize that there are situations where the, e.g., ecology or geographical distribution (or even origin) of a new species is entirely unknown; thinly annotated herbarium specimens come to mind. We are not against species descriptions based on legacy specimens, but a random selection of 100 studies in Suppl. material 2 did not present a single such case. Our dataset presumably still contains examples of this and other special cases, but the vast majority of the studies covered represented relatively recently collected material. This makes the lack of data on ecology and geography harder to swallow.

We were happy to note that the proportion of species descriptions using sequence data is on the rise (Fig. 1). DNA data are important for taxonomic identification in many studies. Failure to provide characters for taxonomic identification using DNA sequencing (typically in the form of sequences deposited in a public repository such as GenBank) is therefore limiting the value of a description. Put negatively, 9.2% of the studies in Suppl. material 2 were done without DNA data. We ask the fungal taxonomists of the world to always bundle at least an ITS sequence (and preferably also an nLSU sequence) with all new species (and genomes, for that matter), even if those sequences were not analyzed or used in the study in question. We recognize that not all mycologists have access to DNA sequencing equipment, and we hope that more well-equipped mycological laboratories will be able to assist nearby, less well-equipped mycologists with the generation of such data. The cost of generating a Sanger sequence is low today, and all of mycology would stand to benefit from such generous acts (cf. Womack 2015).

Adopting a species description to be meaningful also to a non-taxonomic reader may be challenging enough, but we argue that mycological taxonomy needs to go one step further. In a world where information is increasingly culled through automatic means, mycologists should no longer assume that all readers of species descriptions are human to begin with. This means that all data and metadata items should be machine-readable and available online, come with globally unique and persistent identifiers (including ORCIDs for humans, accession numbers/DOIs for sequence data/datasets, DOIs for cited publications, and Open Tree of Life identifiers for phylogenies). The notion of automated readers also brings about changes in the way manuscripts should be written in that it becomes particularly important to provide clear and precise information, almost to the point of tabularization. We argue that standardized terms should be used even when they cannot be parameterized; “Ecology: unknown” is incomparably more helpful to human and automated readers alike than simply leaving out the word “ecology” altogether. Along the same lines, and although brevity may suffer somewhat, “in herbarium GB (University of Gothenburg)” is immeasurably more helpful than “in GB”. No automated reader, and few non-taxonomists, will be able to contextualize the acronym “GB” in a meaningful way. To assume that the reader will be able to extrapolate the position of the species in the fungal tree of life, or the GPS co-ordinates of the collection spot, and so on, should similarly be avoided.

Our demographical estimates suffered from various potential shortcomings and biases: online information can be hard to find (particularly when it comes to authors who have not registered on ORCID), Google Scholar profiles are not necessarily complete or correct in terms of their publication lists, the last author does not have to be a supervisor or a mentor figure, and so on. Getting around these shortcomings in a study of the present kind is next to impossible, and we feel that our demographical results should be seen merely as rough estimates of trends. But a surprisingly strong signal still came out of them: taxonomy is no longer – and perhaps never really was – an entirely solitary discipline, but instead comes across as a reasonably collaborative, international discipline where knowledge seems to be passed on to younger researchers, at least to some extent. This offers hope for the future – taxonomy may actually be on its way to shake some of the misconceptions surrounding it (Coleman 2015). Still, taxonomy needs to be wary of gender aspects as evinced by our estimate that a total of 29.8% of the co-authors in our dataset were female, indicating that a lot of potential competence and perspectives are neglected by the field. There may also be other inequalities in the discipline, not estimated here.

Our data generally, but not exclusively, indicate what we feel is a gradual improvement in the richness of species descriptions and in the demographical aspects of fungal taxonomy over time (Table 2). The improvements come across as slow but at least steady. There is little in our data to invite complacency, though. On the contrary, we feel that our data highlight the need for fungal taxonomy to make a sincere effort to be aware and to improve. It is not enough that mycological species descriptions perhaps come across as a bit more robust and reproducible than plant descriptions in our somewhat compromised comparison. We should set the bar really high – this-and-that is what we want species descriptions to look like from the scientific, demographic, and popular science points of view, so this-and-that is indeed what we will deliver. We feel that there are several measures that fungal taxonomists can and should take to improve the impact and outreach of their work. Our suggestions – with the exception of generating and bundling a DNA sequence with each species description – do not come with any direct costs and do not call for acquisition of any machines or utensils. That said, we don’t want our suggestions to be used as excuses for cutting down other parts of species descriptions – we don’t feel that the inclusion of a macroscopic color photo justifies shortening the corresponding text-based description of the macromorphology, for instance. Our suggestions will add somewhat to the time it takes to describe a new species – or serve as the editor or reviewer of a manuscript in which a new species is described – but we argue that it is a price worth paying (de Carvalho et al. 2008). Indeed, we feel, it is a price that mycology simply must pay to make it a reproducible field at the heart of systematics and biodiversity at large.

## Supplementary materials

Supplementary material 1 Python code responsible for the PDF parsing Data type Source code (PDF) Explanation note The Python code responsible for the PDF parsing, including the full details on how searches were made for each of the entries in Table 2. https://binary.pensoft.net/file/451378This dataset is made available under the Open Database License (http://opendatacommons.org/licenses/odbl/1.0/). The Open Database License (ODbL) is a license agreement intended to allow users to freely share, modify, and use this Dataset while maintaining this same freedom for others, provided that the original source and author(s) are credited.Louisa Durkin, Tobias Jansson, Marisol Sanchez, Maryia Khomich, Martin Ryberg, Erik Kristiansson, R. Henrik Nilsson

Supplementary material 2 The automatic and manual estimates for the targeted mycological papers Data type Raw data (statistics) Explanation note The automatic and manual estimates for each of the 1,097 mycological papers targeted. The corresponding plant data are available on the second sheet. https://binary.pensoft.net/file/451379This dataset is made available under the Open Database License (http://opendatacommons.org/licenses/odbl/1.0/). The Open Database License (ODbL) is a license agreement intended to allow users to freely share, modify, and use this Dataset while maintaining this same freedom for others, provided that the original source and author(s) are credited.Louisa Durkin, Tobias Jansson, Marisol Sanchez, Maryia Khomich, Martin Ryberg, Erik Kristiansson, R. Henrik Nilsson
