# Supplementary material for: When mycologists describe new species, not all relevant information is provided (clearly enough)
Source: MycoKeys. 2020 Sep 10;72:109–28. doi: 10.3897/mycokeys.72.56691 (PMC7498475; doi:10.3897/mycokeys.72.56691)
Supplement: Supplementary material 1 — Python code responsible for the PDF parsing [file mycokeys-72-109-s001.pdf]

```python
# -*- coding: utf-8 -*-
import subprocess
import re
import io

PDFFILES="pdffiles.txt"
PDFPREFIX="pdf/"
TXTPREFIX="txt/"
OUTFILE="matches.xls"

YEARS=("2008", "2009", "2010", "2011", "2012", "2013", "2014", "2015", "2016", "2017", "2018")

PATTERNS={"ECOLOGY_TERM"    :{"ecology"   : r"\b[Ee]colog[A-Za-z]+\b"},
          "ECOLOGY_MODE"    :{"trophic1"  : r"\b[A-Za-z-]*troph[A-Za-z]*\b",
                              "trophic2"  : r"\b[A-Za-z-]+trophic\b",
                              "saprobe"   : r"\bsaprob[A-Za-z]*\b",
                              "mutualis"  : r"\bmutualis[A-Za-z]+\b",
                              "parasite"  : r"\bparasit[A-Za-z]+\b",
                              "commensal" : r"\bcommensa[A-Za-z]+\b",
                              "mycorrhiza": r"\b[A-Za-z-]*mycorrhiz[A-Za-z]+\b",
                              "phyte"     : r"\b[A-Za-z-]+phyte[A-Za-z]*\b",
                              "phytic"    : r"\b[A-Za-z-]+phytic[A-Za-z]*\b",
                              "predator"  : r"\b[A-Za-z-]*predator[A-Za-z]*\b",
                              "predacious": r"\b[A-Za-z-]*predaceous[A-Za-z]*\b",
                              "biont"     : r"\b[A-Za-z-]+biont[A-Za-z]*\b",
                              "pathogen"  : r"\b[A-Za-z-]*pathog[A-Za-z]+\b",
                              "lithic"    : r"\b[A-Za-z-]+lithic[A-Za-z]*\b",
                              "endo"      : r"\b[A-Za-z-]*endo[A-Za-z]+\b"},
          "ECOLOGY_ASSOC"   :{"substrate" : r"\b[A-Za-z-]*substrates*\b",
                              "habitat"   : r"\b[A-Za-z-]*habitats*\b",
                              "host"      : r"\b[A-Za-z-]*hosts*\b",
                              "partner"   : r"\b[A-Za-z-]*partners*\b"},
          "COUNTRY"         :{"country"   : r"\bcountr[A-Za-z-]+\b"},
          "LOCALITY"        :{"local"     : r"\blocalit[A-Za-z-]+\b"},
          "GIS/GPS"         :{"GIS"       : r"\bgis\b",
                              "GPS"       : r"\bgps\b",
                              "coord"     : r"\b[A-Za-z-]+ordinates*\b"},
          "DISTRIBUTION"    :{"geograph"  : r"\bgeograph[A-Za-z]+\b",
                              "dist"      : r"\bdistribut[A-Za-z]+\b"},
          "ALTITUDE"        :{"alt"       : r"\baltitud[A-Za-z]+\b",
                              "ele"       : r"\belevati[A-Za-z]+\b"},
          "CLIMATE_ZONE"    :{"boreal"    : r"\b[A-Za-z-]*boreal\b",
                              "temper"    : r"\b[A-Za-z-]*temperate\b",
                              "arctic"    : r"\b[A-Za-z-]*arctic[A-Za-z]*\b",
                              "tropic"    : r"\b[A-Za-z-]*tropic[A-Za-z]*\b",
                              "africa"    : r"\b[A-Za-z-]*africa[A-Za-z]*\b",
                              "europe"    : r"\b[A-Za-z-]*europe[A-Za-z]*\b",
                              "america"   : r"\b[A-Za-z-]*america[A-Za-z]*\b",
                              "asia"      : r"\b[A-Za-z-]*asia[A-Za-z]*\b",
                              "austral"   : r"\baustral[A-Za-z]*\b"},
          "CLIMATE"         :{"climate"   : r"\bclimat[A-Za-z]+\b"},
          "MOLECULAR_MARKER":{"DNA"       : r"\b[A-Za-z-]*dna\b",
                              "seq"       : r"\bsequenc[A-Za-z]+\b",
                              "PCR"       : r"\b[A-Za-z-]*pcr\b"},
          "MOLECULAR_DB"    :{"genbank"   : r"\bgenbank\b",
                              "INSD"      : r"\binsd\b",
                              "INSDC"     : r"\binsdc\b",
                              "ENA"       : r"\bena\b",
                              "EMBL"      : r"\bembl\b",
                              "DDBJ"      : r"\bddbj\b"},
          "MOLECULAR_AVAIL" :{"treebase"  : r"\btreebase\b",
                              "dryad"     : r"\bdryad\b"},
          "MOLECULAR_BLAST" :{"blast"     : r"\b[tnpx]*blast[tnpx]*\b"},
          "PHYLUM"          :{"mycota"    : r"\b[A-Za-z]+mycota\b"},
          "ORDER"           :{"ales"      : r"\b[A-Za-z]{4,50}ales\b"},
          "FAMILY"          :{"aceae"     : r"\b[A-Za-z]+aceae\b"},
          "BIODIVERSITY"    :{"biodiv"    : r"\bbiodiversity\b"},
          "THREATEND"       :{"threat"    : r"\bthreatened\b",
                              "end"       : r"\bendangered\b"},
```

```
           "COLLECTION"      :{"herb"      : r"\bherbari[A-Za-z]+\b",
                               "fung"      : r"\bfungari[A-Za-z]+\b",
                               "museum"    : r"\bmuseum[A-Za-z]*\b",
                               "collection": r"\bculture collection[s]*\b"},
           "SUPPLEMENTAL"    :{"supp1"     : r"\bsupplementa[A-Za-z]+\b"},
           "INDEX_FUNGORUM"  :{"if"        : r"\bindex fungorum\b",
                               "iflink"    : r"indexfungorum"},
           "MYCOBANK"        :{"mb"        : r"\bmycobank\b"},
           "FUNGAL_NAMES"    :{"fn"        : r"\bfungal names\b"},
           "SOCIETAL_IMPL"   :{"agri"      : r"\bagricultur[A-Za-z]+\b",
                               "for"       : r"\bforestry\b",
                               "biotech"   : r"\bbiotechnolog[A-Za-z]+\b",
                               "med"       : r"\bmedic[A-Za-z]+\b",
                               "clinic"    : r"\bclinic[A-Za-z]+\b",
                               "health"    : r"\bhealth *care\b",
                               "ind"       : r"\bindustr[A-Za-z]+\b",
                               "aqua"      : r"\baquacultur[A-Za-z]+\b",
                               "horti"     : r"\bhorticultur[A-Za-z]+\b"}}

EXCLUDEDPATTERNS={"ECOLOGY_MODE": {"trophic1"  : r"electrophor",
                                   "endo"      : r"eppendorf"}}


def readPDFList(PDFFILE):

    rawdata=open(PDFFILE).readlines()

    pdffiles=[]

    for line in rawdata:
        pdffiles.append(line.strip())

    return pdffiles


def convertPDFtoTXT(pdffiles):

    txtfiles=[]

    for pdffile in pdffiles:

        print "Converting %s" % pdffile

        txtfile=pdffile+".txt"
        txtfile_old=pdffile+"-old.txt"

        process=subprocess.Popen(["pdftotext", PDFPREFIX+pdffile, TXTPREFIX+txtfile])
        stdout, stderr = process.communicate()

        process=subprocess.Popen(["pdftotext", "-raw", PDFPREFIX+pdffile, TXTPREFIX+txtfile_old])
        stdout, stderr = process.communicate()

        txtfiles.append(txtfile)

    return txtfiles

def processArticles(txtlist):

    all_matches={}

    for txtfile in txtlist:
        print "Parsing %s" % txtfile
        all_matches[txtfile]=parseArticle(TXTPREFIX+txtfile)

    return all_matches

def parseArticle(txtfile):

```

```
    matches={}
    references_found=0
    abstract_found=0
    acknowledgements_found=0
    funding_found=0

    print "Parsing %s" % txtfile

    indata=open(txtfile).readlines()

    cntr_start=0

    # Search for year

    # This code search for a year associated with several key phrases
    # Exceptions is made for Mycologia since those PDFs often contains an additional page where the
    # available online date can differ significantly from the published date. Also, in some Mycologia
    # papers, the years appears on the same line and for some the year appears on the line below.
    # The loop continutes until a year has been found. If no year is found, the value will be -1.

    myco_year_on_next_line=0
    is_mycologia=0
    year=-1

    # Check first line. Some papers have spaces between the numbers.
    tmp=indata[0].replace(" ", "")
    for yr in YEARS:
        if yr in tmp:
            year=yr
            break

    # Continue if no year was found.
    if year==-1:
        for row in indata:
            row=row.strip().lstrip().lower()

            if year!=-1:
                break

            if myco_year_on_next_line==1:
                for yr in YEARS:
                    if yr in row:
                        year=yr
                        break

            if row[:21]=="to cite this article":
                for yr in YEARS:
                    if yr in row:
                        year=yr
                        break

            if "accepted:" in row:
                tmp=row.split("accepted:")[-1].split(" ")[-1].strip()
                if tmp in YEARS:
                    year=tmp
                    break

            if row[:16]=="available online" and is_mycologia==0:
                for yr in YEARS:
                    if yr in row:
                        year=yr
                        break

            if row[:9]=="mycologia":
                is_mycologia=1
```

```
                for yr in YEARS:
                    if yr in row:
                        year=yr
                        break
                if year==-1:
                    myco_year_on_next_line=1

    if year==-1:
        print "Warning: Year not found."
    else:
        print "Found year %s" % year

    matches["year"]=year

    # Search for abstract
    for row in indata:

        row=row.strip().lower()
        row=row.replace(" ", "")

        if row=="abstract" or row=="a b s t r a c t" or row[:8]=="abstract" or row[:15]=="a b s t r a c t":
            abstract_found=1
            print "Found abstract at row %d" % cntr_start
            break

        cntr_start+=1

    if abstract_found==0:
        print "Warning: No abstract found."

    cntr=0
    tot_row=len(indata)
    for row in indata:

        if cntr>=cntr_start and (("the mycological society of america" not in row.lower()) or ("copyright" not in row.lower())):
            row=row.strip().lower()
            for section in PATTERNS:
                for pattern in PATTERNS[section]:
                    foundmatch=0
                    if re.search(PATTERNS[section][pattern], row)!=None:
                        if section in EXCLUDEDPATTERNS and pattern in EXCLUDEDPATTERNS[section]:
                            if re.search(EXCLUDEDPATTERNS[section][pattern], row)==None:
                                matches[section]=1
                                foundmatch=1
                        else:
                            matches[section]=1
                            foundmatch=1
                    if foundmatch==1:
                        print "Found %s (%s, %s) in %s." % (pattern, section, PATTERNS[section][pattern], row)



#               print 1.0*cntr/tot_row, row
            if (((1.0*cntr)/tot_row)>0.50 and (row=="references" or row=="r e f e r e n c e s" or row=="literature cited" or row=="l i t e r a t u r e   c i t e d" or row=="reference" or row=="list of references")):
                references_found=1
                break

            if (((1.0*cntr)/tot_row)>0.50 and (row=="acknowledgements" or row=="a c k n o w l e d g e m e n t s" or row=="acknowledgments" or row=="a c k n o w l e d g m e n t s" or row[:16]=="acknowledgements" or row[:15]=="acknowledgments")):
                acknowledgements_found=1
                break

```

```python
            if (((1.0*cntr)/tot_row)>0.50 and (row=="funding" or row=="f u n d i n g")):
                funding_found=1
                break


        cntr+=1

    if acknowledgements_found==1:
        print "Exited at acknowledgements (row %d of %d)" % (cntr, tot_row)
    else:
        print "No acknowledgements found"

    if references_found==0:
        print "Warning: No references found!"
    else:
        print "Exited at reference (row %d of %d)" % (cntr, tot_row)

    if funding_found==0:
        print "Warning: No funding found!"
    else:
        print "Existed at funding (row %d or %d)" % (cntr, tot_row)

    print "Done!\n\n"
    return matches

def writeResults(matches):

    colnames=sorted(PATTERNS.keys())
#    print colnames

    of=open(OUTFILE, "w")

    of.write("Papers\tJournal\tYear\t%s\n" % "\t".join(colnames))

    for paper in matches:
        curline=[]
        for colname in colnames:
            if colname in matches[paper]:
                curline.append("Y")
            else:
                curline.append("N")

        tmp=paper.split("/")
        journal=tmp[-2]
        article=tmp[-1]

        of.write("%s\t%s\t%s\t%s\n" % (article, journal, matches[paper]["year"],"\t".join(curline)))

    of.close()

    return





pdflist=readPDFList(PDFFILES)
txtlist=convertPDFtoTXT(pdflist)
#txtlist=["warpeace.txt"]
matches=processArticles(txtlist)
writeResults(matches)
```
